# Food, Feed, and Phytochemical Uses of Wild Edible Plants: A Systematic Review

**DOI:** 10.1002/fsn3.70454

**Published:** 2025-06-19

**Authors:** Tamene Daba Rumicha, Sileshi Belew, Gemmechu Hasen, Tilahun A. Teka, Sirawdink Fikreyesus Forsido

**Affiliations:** ^1^ Department of Postharvest Management, College of Agriculture and Veterinary Medicine Jimma University Jimma Ethiopia; ^2^ Department of Food and Nutritional Sciences, Faculty of Agriculture Wollega University Shambu Ethiopia; ^3^ School of Pharmacy Jimma University Jimma Ethiopia

**Keywords:** biodiversity, ethnobotony, functional foods, nutraceuticals, nutritional composition, phylogenetic, wild edible plants

## Abstract

Wild edible plants (WEPs) are potential food, feed, and health benefit sources. The present study describes a comprehensive synthesis on the food, feed, and pharmaceutical uses of WEPs distributed in different parts of Ethiopia. A systematic literature search strategy retrieved 689 articles from PubMed and Semantic Scholar electronic databases. The online search of articles was conducted from January to April 2023. The retrieved articles were screened against inclusion criteria. Finally, 129 full‐text articles complying with eligibility criteria were included. The results of the reviewed articles revealed that there are 679 WEP species registered in Ethiopia, and the distribution of WEPs among different regions of the country is widely different. The results indicated that various aspects of 286 WEP species were described in at least two or more pieces of literature. Though there are differences in modes of consumption and preparation across communities living in different regions of Ethiopia, there is a similar tradition of consuming fruits, leaves, roots, young stems, and whole parts of WEPs for food, feed, and/or health benefit purposes. WEP families (i.e., Fabaceae, Moraceae, and Malvaceae) and species (i.e., *Syzygium guineense, Cordia africana, Carissa spinarum*, and *Ficus sur*) were reported to be widely consumed by different communities. Shrubs were reported as major plant growth forms, followed by trees, herbs, and climbers. The results indicated that WEPs have rich potential in Ethiopia. Thus, investigating the food, feed, and pharmaceutical aspects of WEPs is vital to alleviate food security challenges and explore alternative sources of pharmaceutical ingredients.

## Graphical abstract

There are approximately 679 wild edible plant species in Ethiopia, of which 286 are commonly consumed across different regions, indicating a diverse range of wild edible plant sources. Wild edible plants in Ethiopia are utilized for food, animal feed, and pharmaceutical purposes by communities across the country. Despite the diverse potential of wild edible plants (WEPs) in Ethiopia, information on their nutritional and bioactive compounds is limited to only a small number of species.

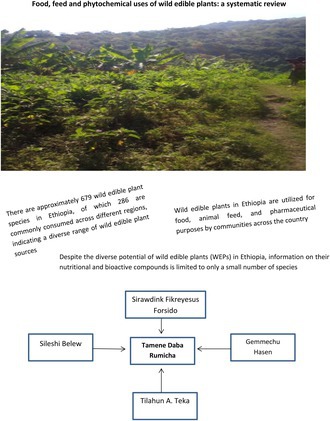

## 1 Introduction

Food security is an ongoing global challenge influencing the well‐being and health of millions of people. The problem is vast in Sub‐Saharan Africa and Asia (Chichaibelu et al. 2021; FAO 2021). Wild edible plants (WEPs) are natural gifts that can be one of the best alternative resources to combat global food security and health challenges. However, so far, the use of WEPs to ensure the food security problem is minimal, and the tradition of consuming WEPs for food/feed, health benefits, and commercial purposes is localized to some community members/society (Delang 2006). Thus, exploring the potential of WEPs and promoting WEPs as an alternative source of food, feed, and/or pharmaceuticals is vital.

WEPs play a critical role in enhancing global food security and promoting climate‐resilient agriculture. These underutilized species often thrive in marginal environments, making them vital for communities vulnerable to climate variability and food system disruptions (Bharucha and Pretty 2010). WEPs contribute significantly to dietary diversity, providing essential micronutrients, vitamins, and minerals, especially for rural and indigenous populations (Hunter et al. 2019). Their adaptation to local agroecological conditions often requires minimal inputs, such as irrigation or synthetic fertilizers, making them inherently resilient to drought, poor soils, and pests (Heywood 2011). Furthermore, the ecological roles of WEPs such as soil stabilization, pollinator support, and biodiversity conservation align closely with the principles of sustainable and climate‐smart agriculture (Padulosi et al. 2013). Globally, the recognition and integration of WEPs into agricultural policy and research agendas can diversify food systems, reduce dependence on a narrow set of staple crops, and increase the resilience of livelihoods to environmental and economic shocks (Toledo and Burlingame 2006). Promoting the sustainable use and conservation of WEPs offers a strategic pathway toward achieving multiple sustainable development goals (SDGs), including zero hunger (SDG 2), good health and well‐being (SDG 3), and climate action (SDG 13).

WEPs have long been an integral part of human survival and cultural heritage, serving as essential sources of food, feed, and medicinal remedies worldwide. These plants, naturally occurring in forests, grasslands, and uncultivated areas, represent a critical component of biodiversity and play a significant role in ensuring food and nutritional security, especially in rural and indigenous communities (Bharucha and Pretty 2010). In many traditional societies, WEPs contribute substantially to daily diets, particularly during periods of food scarcity or crop failure, acting as a buffer against hunger and malnutrition (Heywood 2011).

In addition to their role as human food, WEPs are vital for animal feed, particularly in traditional agro‐pastoral systems. Many wild species serve as fodder for livestock, contributing to sustainable livestock production and reducing the dependence on commercial feed resources (FAO 2019). Beyond their nutritional value, WEPs possess a range of bioactive compounds with medicinal properties, including anti‐inflammatory, antimicrobial, antioxidant, and antidiabetic effects (Afolayan and Jimoh 2009). Their use in traditional medicine highlights their potential for developing functional foods and nutraceutical products aimed at promoting health and preventing disease (Wachtel‐Galor 2011).

Ethiopia is one of the sub‐Saharan countries with rich biodiversity. It is the habitat for more than 651 species of WEPs identified so far (Tadesse et al. 2024). However, most WEPs are not characterized and are utilized as a source of food, feed, pharmaceuticals, and other health‐promoting functions. Though WEPs are one of the potential sources that cannot be disregarded (Bvenura and Afolayan 2015), due attention has not been given to the expected level. Therefore, exploring the literature and identifying the prevailing research gaps help exploit WEPs to ensure food security and improve public health and commercialization. This study presents comprehensive literature describing the food, feed, and/or pharmaceutical uses of WEPs distributed in different parts of Ethiopia.

WEPs serve diverse purposes across food, feed, and pharmaceutical sectors, each characterized by distinct uses and target beneficiaries. In the context of food, WEPs are primarily consumed by humans for their nutritional benefits. They are often rich in essential nutrients such as vitamins, minerals, fiber, and antioxidants. These plants play a vital role in traditional diets and contribute significantly to food security, especially in rural and indigenous communities. For instance, species like *Amaranthus* spp., *Portulaca oleracea* (purslane), and *Moringa oleifera* are commonly used as vegetables or herbs due to their high nutritional content and adaptability to various environments (Bharucha and Pretty 2010).

In contrast, when used as feed, WEPs serve as fodder for livestock, providing essential nutrients needed for animal growth, health, and productivity. These plants are especially important in regions where conventional animal feed is scarce or expensive. They include species such as *Leucaena leucocephala* , *Acacia* spp., and *Opuntia* spp., which are consumed directly by grazing animals or harvested for silage and hay. The utilization of such plants can reduce feeding costs and support sustainable livestock systems in both arid and semi‐arid regions (Franzel et al. 2014).

Meanwhile, in the pharmaceutical domain, WEPs are valued for their therapeutic properties and bioactive compounds such as alkaloids, flavonoids, phenolics, and terpenoids. These compounds have been traditionally used to treat various ailments and are currently being researched for modern drug development. Examples include *Centella asiatica* , known for cognitive and skin healing benefits, *Withania somnifera* (ashwagandha), recognized for its adaptogenic properties, and *Garcinia kola* , used in traditional medicine for treating coughs and infections. The pharmaceutical application of WEPs underscores their significance beyond nutrition, highlighting their role in ethnomedicine and pharmacology (Pullaiah et al. 2017; Van Wyk 2017).

The review was aimed to fill the research gap in exploring comprehensive data on WEPs and their use in the context of Ethiopia which is critical to capture the various aspects of WEPs and pave the way forward in expanding the efficient utilization strategies and preservation approaches of WEPs. This review is also important in recognizing the role of WEPs within global knowledge systems and policy frameworks, particularly those of the Food and Agriculture Organization of the United Nations (FAO), in promoting sustainable food systems, biodiversity conservation, and climate adaptation. The FAO has emphasized the significance of WEPs in several key initiatives, including the State of the World's Biodiversity for Food and Agriculture (FAO 2019), which highlights wild food plants as vital to the resilience of local food systems and the preservation of cultural traditions. Additionally, through the Global Plan of Action for the Conservation and Sustainable Use of Plant Genetic Resources for Food and Agriculture (FAO 2011), the FAO encourages member states to document, conserve, and promote the sustainable use of WEPs.

## 2 Methods

### 2.1 Data Sources and Search Strategy

PubMed and Semantic Scholar databases/search engines were used to retrieve original articles. The keywords, i.e., Wild edible plants AND (nutrition OR antioxidant OR toxicity OR feed OR phytochemical OR anti‐nutritional factors OR Ethiopia OR food security) were used as search terms.

### 2.2 Selection of Articles and Data Extraction

Original articles written in English and published between 2000 and 2023 that describe food, feed, and/or pharmaceutical use of WEPs in Ethiopia were included. In contrast, articles that do not describe food, feed, and pharmaceutical uses of WEPs and review articles were excluded. Qualified and experienced reviewers (*n* = 3) carefully reviewed retrieved articles' titles, abstracts, and full text. Finally, relevant data were extracted from those articles complying with eligibility criteria. The review of the retrieved articles was conducted with no significant disagreement in the selection of articles and extraction of relevant information. The minor disagreements were resolved considering the objectives of the review, credibility, and quality of the studies.

The articles included in this review were systematically retrieved in accordance with the PRISMA (Preferred Reporting Items for Systematic Reviews and Meta‐Analyses) guidelines, ensuring a transparent and reproducible selection process. A PRISMA flowchart was utilized to visually represent the identification, screening, eligibility, and inclusion phases of the study selection, providing a clear overview of how the final set of studies was determined. To assess the methodological quality and risk of bias in the included studies, the Joanna Briggs Institute (JBI) quality appraisal criteria were applied. The JBI tool comprises nine standardized evaluation checklists, each tailored to different study designs, and is widely recognized for its rigor in systematic reviews. These criteria evaluate key aspects of study quality, including the appropriateness of the study design and setting, the time period during which the research was conducted, and the suitability of the sampling frame. Additionally, they assess the process for selecting study participants, the adequacy and representativeness of the sample size, and whether the data analysis provided sufficient coverage of the identified sample. The criteria also examine the validity and reliability of the outcome measurements, the consistency and standardization of measurement methods across participants, and the appropriateness of the statistical analysis used. Furthermore, it was assessed whether the studies adequately addressed potential confounding factors through appropriate design or analytical strategies. The use of these comprehensive quality appraisal tools ensured that only methodologically sound and credible studies were included in the synthesis.

During the review process, inter‐reviewer agreement statistics, such as Cohen's kappa, were used to assess the level of agreement between reviewers during article selection and data extraction. By accounting for the agreement expected by chance, this statistic provides a more robust measure of consistency than simple percentage agreement. Incorporating such metrics in systematic reviews enhances methodological transparency and strengthens the reproducibility and credibility of the selection process.

## 3 Results

### 3.1 Search Results

**FIGURE 1:**
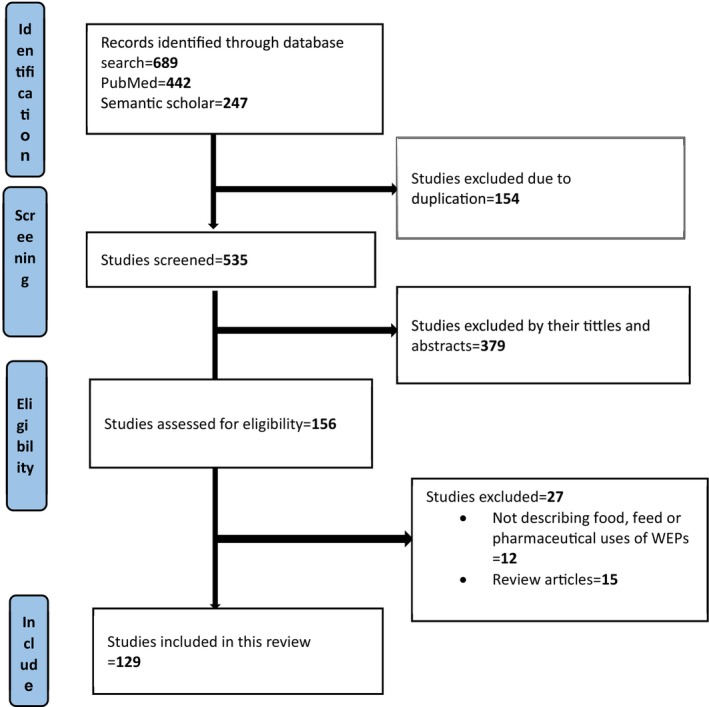
Flow diagram that shows the search and selection process.

689 articles were retrieved from PubMed and Semantic Scholar electronic databases. After duplicate (*n* = 154) removal using the Mendeley citation manager, 535 articles were screened by their titles and abstracts. Of these, 379 articles were excluded; the remaining 156 were further assessed for eligibility. Finally, 129 articles were eligible and included in this study. Detailed information on the search and selection process is presented in Figure 1.

**FIGURE 2:**
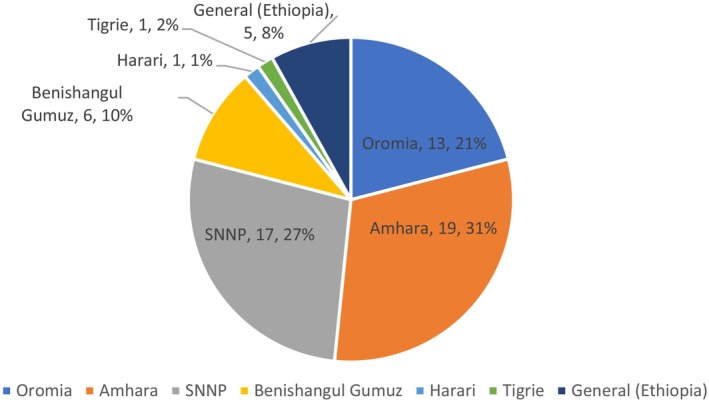
The number and proportion of studies conducted in Ethiopia that describe different aspects of WEPs.

The reviewed literature indicated that WEPs were described in 129 studies conducted worldwide in different geographical regions. These studies investigated different aspects of WEPs, such as ethnobotanical, food, feed, and medicinal uses. Figure 2 shows the number of studies conducted in different regions of Ethiopia.

### 3.2 Ecological and Phylogenetic Distribution of WEPs in Ethiopia

#### 3.2.1 Ecological Distribution

Retrieved articles indicated that 74 families of WEPs comprising 679 species were reportedly used for food, feed, and/or pharmaceuticals. Different aspects of 284 species were described in at least two or more literature. Fabaceae, Moraceae, and Malvaceae, respectively, were reported as the leading WEP families comprising a relatively large number of species (Fabaceae: *n* = 22, Moraceae: *n* = 17, and Malvaceae: *n* = 16) serving as food, feed, and/or pharmaceuticals sources. Communities in all regions of the country consume fruit and leaf of *Syzygium Guineense* for food, feed, and health benefit (Demise 2020; Hankiso et al. 2023; Tahir et al. 2023; Nigatu et al. 2018; Hassen 2021; Ayele and Negasa 2017; Dejene et al. 2020). Similarly, the fruit and leaf of *Cordia Africana*, the fruit, leaf, and root of *Carissa spinarum*, and the fruit of *Ficus sur* were found to be used for food, health benefit, and/or animal feed in a relatively wider region of the country (Mekuanent et al. 2015; Masresha et al. 2023; Guzo et al. 2023; Ayele and Negasa 2017; Feyssa et al. 2011a). Detailed information on WEPs family and species commonly consumed in Ethiopia is presented in Table 1.

**TABLE 1:** List of WEP families with their respective number of species commonly consumed in Ethiopia.

#	Name of families	Number of species	#	Name of family	Number of species
1	Fabaceae	22	38	Brassicaceae	2
2	Moraceae	17	39	Clusiaceae	2
3	Malvaceae	16	40	Dioscoreaceae	2
4	Asteraceae	11	41	Moringaceae	2
5	Amaranthaceae	10	42	Myrtaceae	2
6	Anacardiacaea	10	43	Olacaceae	2
7	Solanaceae	10	44	Oleaceae	2
8	Euphorbiaceae	9	45	Passifloraceae	2
9	Cucurbitaceae	8	46	Plumbaginaceae	2
10	Lamiaceae	8	47	Portulaceae	2
11	Rutaceae	8	48	Salvadoraceae	2
12	Acanthaceae	7	49	Sapotaceae	2
13	Apocynaceae	7	50	Alliaceae	1
14	Poaceae	7	51	Asparagaceae	1
15	Rhamnaceae	6	52	Bignoniaceae	1
16	Rosaceae	6	53	Bolentaceae	1
17	Rubiaceae	6	54	Cactaceae	1
18	Boraginaceae	5	55	Capparaceae	1
19	Polygalaceae	5	56	Caryophyllaceae	1
20	Asclepiadaceae	4	57	Cleomaceae	1
21	Capparidaceae	4	58	Crassulaceae	1
22	Combretaceae	4	59	Cyperaceae	1
23	Ebenaceae	4	60	Juniperus procera	1
24	Flacourtiaceae	4	61	Melianthaceae	1
25	Sapindaceae	4	62	Musaceae	1
26	Tiliaceae	4	63	Myrsinaceae	1
27	Apiaceae	3	64	Oliniaceae	1
28	Araceae	3	65	Oxalidaceae	1
29	Arecaceae	3	66	Papilionaceae	1
30	Burseraceae	3	67	Phyllanthaceae	1
31	Celasteraceae	3	68	Pittosporaceae	1
32	Commelinaceae	3	69	Primulaceae	1
33	Loganiaceae	3	70	Santalaceae	1
34	Menispermaceae	3	71	Ulmaceae	1
35	Verbenaceae	3	72	Urticaceae	1
36	Annonaceae	2	73	Vitaceae	1
37	Balanitaceae	2	74	Zingiberaceae	1

Ethiopia's diverse ecological zones range from high‐altitude plateaus and temperate forests to semi‐arid lowlands and riverine ecosystems. This environmental variation supports WEPs, each adapted to specific climatic and soil conditions. In the highland areas, which are above 2500 m, species tend to adapt to cooler temperatures and higher rainfall. The dominant plant families in this zone include Rosaceae, Poaceae, Rutaceae, and Fabaceae, with species such as *Rubus steudneri* (wild raspberry), *Urtica simensis* (nettle), and *Juniperus procera* thriving in the mountain forests. These species provide fruits, leafy greens, and edible seeds, valuable in traditional Ethiopian diets.

Between 1500 and 2500 m, the mid‐altitude regions are characterized by mountain forests, woodlands, and transitional zones between the highlands and lowlands. This zone hosts many WEPs, including species from families such as Fabaceae, Moraceae, Solanaceae, and Asteraceae. These plants, such as *Ficus sur* (wild fig), *Cordia africana* (large‐leaved cordia), and *Solanum nigrum* (black nightshade), are well‐suited to Ethiopia's seasonally dry climate. These mid‐altitude areas support a mix of fruiting trees, shrubs, and herbaceous species, which contribute to the food security of local communities by providing seasonal food sources.

In the lowland areas, which include arid and semi‐arid regions below 1500 m, the vegetation consists of drought‐resistant species that have adapted to extreme temperature fluctuations and low water availability. Some of the dominant families in these zones include Anacardiaceae, Cucurbitaceae, and Balanitaceae, which are known for their ability to store water and withstand dry conditions. Species such as *Balanites aegyptiaca* (desert date), *Tamarindus indica* (tamarind), and *Salvadora persica* (toothbrush tree) are commonly found in these ecosystems. These species provide fruits, nuts, and edible leaves, playing a crucial role in the diets of pastoralist and agro‐pastoralist communities, especially during periods of food scarcity.

In addition to the highlands, mid‐altitudes, and lowlands, riverine and wetland ecosystems support their own unique WEPs. Along Ethiopia's major rivers and wetlands, species from families such as Araceae, Arecaceae, and Musaceae thrive due to the availability of water and nutrient‐rich soils. Notable examples include *Colocasia esculenta* (taro), *Phoenix reclinata* (wild date palm), and *Ensete ventricosum* (false banana). These species are crucial for communities near rivers and lakes, providing staple food sources and nutritional diversity.

#### 3.2.2 Phylogenetic Distribution

The WEPs found in Ethiopia represent a wide range of plant families, reflecting their diverse evolutionary history and ecological adaptations. Fabaceae is the most dominant family among Ethiopia's WEPs, which includes 22 species. This family is widely distributed across different ecological zones, from the dry lowlands to the moist highlands. Leguminous plants such as *Acacia* spp., *Cajanus cajan* (pigeon pea), and *Vigna* spp. are well known for their ability to fix nitrogen, enhancing soil fertility and making them crucial for agriculture and wild ecosystems.

Another significant family is Moraceae, which includes 17 species. This family comprises various fig species (*Ficus* spp.) commonly found in forests and savannas. Fig trees are ecologically important as they provide food for wildlife and humans alike and play a role in soil conservation. Similarly, Malvaceae, with 16 species, includes trees like *Adansonia digitata* (baobab), which is known for its nutrient‐rich fruit pulp and high drought resistance.

Several other families contribute to the diversity of WEPs in Ethiopia, including Asteraceae, Solanaceae, and Amaranthaceae, which are represented by multiple herbaceous species. These families are often found in disturbed habitats such as roadsides, grasslands, and farmlands. Plants from these families, including *Amaranthus* spp., *Bidens pilosa* , and *Solanum nigrum* , are commonly harvested as leafy greens and medicinal herbs. Their ability to thrive in various environments makes them an important food source in urban and rural settings.

Though represented by fewer species, some families have high ecological and cultural significance. For example, Zingiberaceae, which includes the ginger family, and Dioscoreaceae, which includes yams, are mainly found in moist tropical forests. These species are traditionally used for both food and medicinal purposes. Similarly, the Myrtaceae family, which includes species such as *Syzygium guineense* (wild cherry), is known for its edible fruits and medicinal properties.

Ethiopia's ecological and phylogenetic distribution of WEPs reflects the country's rich biodiversity and environmental diversity. Different ecological zones, ranging from high‐altitude forests to dry lowland savannas, support specific plant families adapting to their respective plant growth forms. The dominance of Fabaceae, Moraceae, and Malvaceae highlights the importance of leguminous plants, fruit‐bearing trees, and drought‐resistant species in Ethiopian diets. Furthermore, the presence of species from a wide range of families, including herbaceous, shrubby, and tree species, demonstrates the evolutionary diversity of Ethiopia's flora. Understanding these distributions is essential for the conservation and sustainable use of WEPs, ensuring they continue to provide nutrition and ecological benefits for future generations.

### 3.3 WEPs Used as Food, Feed, and Health Benefits in Ethiopia

**FIGURE 3:**
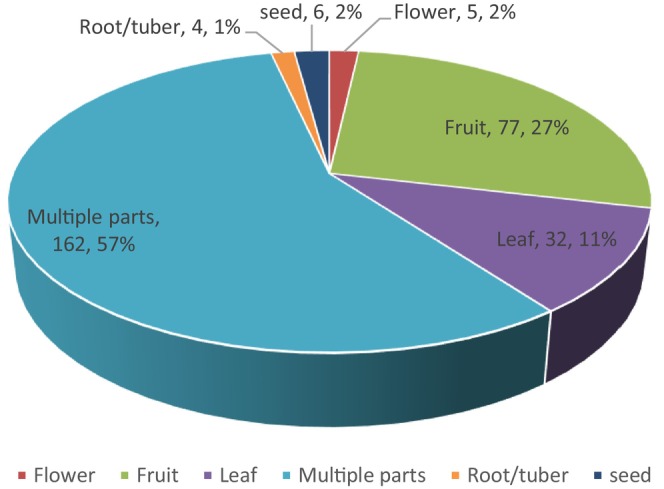
The proportion of WEPs and the number of species having edible parts.

The reviewed literature indicates that various parts of WEPs are consumed in Ethiopia, including leaves, fruits, roots/tubers, flowers, seeds, young stems, gums, bulbs, and barks. Among these, multiple plant parts are consumed from 57% of WEP species, representing 162 species. Fruits account for 27% of WEPs (77 species), while leaves are consumed from 11% of WEPs (32 species). These edible parts are widely used across different communities in the country. Figure 3 illustrates the proportions of WEPs and the number of species with edible parts.

#### 3.3.1 Multifunctional WEP Parts in Different Regions of Ethiopia

The literature indicated that communities in different locations of Ethiopia use parts of WEPs for various purposes, as described below. The leaf and fruit of *Acokanthera schimperi* are used for food and health benefits in the Hadiya Zone of Southern Ethiopia (Hankiso et al. 2023), in Konso, Southern Ethiopia (Addis et al. 2013a); Dhera town of Arsi Zone, Oromia (Wondimu et al. 2006), and in Berehet District, Nort Shewa, Ethiopia (Alemayehu et al. 2015).

The flower of *Acanthus sennii* are consumed for food in the Tach Gayint District of Amhara Region (Yiblet and Adamu 2023), Berehet District, Nort Shewa, Amhara, Ethiopia (Alemayehu et al. 2015), and Bullen District of Northwestern Ethiopia (Berihun and Molla 2017). In addition, leaves of *Acanthus sennii* are reported to be used for food in the Hadiya Zone of Southern Ethiopia (Hankiso et al. 2023) and for medicinal purposes in the District of Libo Kemkem, Northwestern Ethiopia (Getnet et al. 2015).

The leaves and young stems of *Amaranthus caudata* are used for food in Bullen District of North West Ethiopia (Berihun and Molla 2017), Derashe and Kucha Districts, South Ethiopia (Balemie and Kebebew 2006), and Adola, Southern Ethiopia (Demise 2020), and for health benefits in the Hadiya Zone of Southern Ethiopia (Hankiso et al. 2023) and Chelia District, West‐Central Ethiopia (Regassa et al. 2015).

The leaves of *Aloe camperi schweif* are used for food and health benefit in Delanta, Amhara, Ethiopia (Meragiaw et al. 2015), and Dalle District, Sidama Zone, Ethiopia (Nigatu et al. 2018), and its flower is also used for food and health benefit in Ensaro, North Shewa, Amhara (Asfaw et al. 2022), and in Konso, Southern Ethiopia (Addis et al. 2013a) and its root for medicinal purposes in the District of Libo Kemkem, Northwest Ethiopia (Getnet et al. 2015).

The leaf of *Anethum graveolens* is eaten as a food in the districts of Bullen, Northwest Ethiopia (Berihun and Molla 2017), and its flowers in Kamashi, Benishangul Gumuz (Amente 2017). The fruit of *Annona senegalensis* is used for food in Kamashi, Benishangul Gumuz (Amente 2017), Maale and Ari Ethnic, Southern Ethiopia (Kidane et al. 2014), and Bullen District of Northwest Ethiopia (Berihun and Molla 2017) and its root is used for food in Menge Woreda, Benishangul Gumuz region of Ethiopia (Ayele and Negasa 2017).

The leaf of *Asparagus africanus* is used for food in the Hadiya Zone of Southern Ethiopia (Hankiso et al. 2023). In contrast, its root has a health benefit for the treatment of different diseases in Dalle District, Sidama Zone, Ethiopia (Nigatu et al. 2018), Soro District of Hadiya Zone, Southern Ethiopia (Hankiso et al. 2023), and Libo Kemkem District of Northwestern Ethiopia (Getnet et al. 2015).

The fruit of *Balanites aegyptiaca* is used for food, feed, and health benefits in Miesso, Hararge, and Oromia (Tahir et al. 2023). Its leaf and flower are consumed for food in Konso, Southern Ethiopia (Addis et al. 2013a), and Menge Woreda, Benishangul Gumuz region of Ethiopia (Ayele and Negasa 2017). The leaves of *Bidens macroptera* are used for food/health benefits in Meinit, Bench‐Maji, Southern Ethiopia (Yimer et al. 2023c), and its flowers are also used for health benefits in the Libo Kemkem District of Northwestern Ethiopia (Getnet et al. 2015).

Different parts of *Boletus edulis* are used for food, i.e., fruit in Quara district, Northwest Ethiopia (Tebkew et al. 2018), stem in Chilga District, Northwestern Ethiopia (Mekuanent et al. 2015), its leaves in Konso, Southern Ethiopia (Dechassa et al. 2012), and its whole part in Adola, Southern Ethiopia (Demise 2020). The fruit of *Bridelia scleroneura* is consumed for food in the Bullen District of Northwestern Ethiopia (Berihun and Molla 2017) and Konso, Southern Ethiopia (Addis et al. 2013a). In contrast, its leaf is used for food/health benefits in the Soro District of Hadiya Zone, Southern Ethiopia (Hankiso et al. 2023).

The fruit/root of *Cadaba farinosa* is used for food/health benefits in the Konso, Southern Ethiopia (Dechassa et al. 2012), and its leaf for food in Kara and Kwego, Omo Valley, Southern Ethiopia (Teklehaymanot and Giday 2010), and a community around Nech Sar National Park, Ethiopia (Leta 2016).

The Fruit of *Carissa spinarium* is eaten for food/health benefit in Bullen District Northwest Ethiopia (Berihun and Molla 2017), Ensaro, North Shewa, Amhara (Asfaw et al. 2022), Meinit, Bench‐Maji, Southern Ethiopia (Yimer et al. 2023c), Chilga District, Northwestern Ethiopia (Mekuanent et al. 2014), and its leaf is used for food/health benefit in Delanta, Amhara, Ethiopia (Meragiaw et al. 2015), Menge Woreda, Benishangul Gumuz region of Ethiopia (Ayele and Negasa 2017). In contrast, its root is also used for medicinal purposes in Chelia District, West‐Central Ethiopia (Regassa et al. 2015), Dheeraa town, Arsi Zone, Oromia Regional State of Ethiopia (Wondimu et al. 2006), and Libo Kemkem District, Northwestern Ethiopia (Getnet et al. 2015).

The fruit of *Chasmanthera dependens* is used for food in Konso, Southern Ethiopia (Getachew et al. 2013), Borana Pastoralists in Southern Oromia, Ethiopia (Gemedo‐Dalle et al. 2005), and its root/leaf is used for food/health benefit in Borana Pastoralists in Southern Oromia, Ethiopia (Gemedo‐Dalle et al. 2005), and South Omo, Southern Ethiopia (Ketema et al. 2013).

The root of *Ceropegia cunfodontis* is consumed as food in Konso, Southern Ethiopia (Addis et al. 2013a), while its seed is used for food and health benefits in Delanta, Amhara Region (Meragiaw et al. 2015). The fruit of *Clutia lanceolata* is eaten by communities in Bullen District, Northwestern Ethiopia (Berihun and Molla, 2017), and its root is used for medicinal purposes in Libo Kemkem District, also in Northwestern Ethiopia (Getnet et al. 2015). The tuber of *Coccinia abyssinica* is consumed as food in North Wollo, Ethiopia (Hassen 2021), and its leaf is also used as food in Konso, Southern Ethiopia (Addis et al. 2013a).

The fruit of *Coccinia grandis* is consumed as food in Kara and Kwego, Omo Valley, Southern Ethiopia (Teklehaymanot and Giday 2010), and North Wollo, Ethiopia (Hassen 2021). In contrast, its leaf is also used for food in Konso, Southern Ethiopia (Addis et al. 2013a).

The leaf of *Commelina Benghalensis* is used for food or health benefits in the Soro District of Hadiya Zone, southern Ethiopia (Hankiso et al. 2023) and Meinit, Bench‐Maji, Southern Ethiopia (Yimer et al. 2023c), and its root is also consumed as a food in Chelia District, West‐Central Ethiopia (Regassa et al. 2015).

The reviewed literature also indicated that different societies in different regions of the country consume 35 WEP species for food, feed, and health benefit purposes. Detailed information on WEPs used for food, feed, and health benefits is presented in Table 2.

**TABLE 2:** List of WEPs families and species used for food, feed, and/or health benefit.

No.	Plant species	Family	Part used	Plant growth forms	Use of plant	References
1	*Acacia tortilis* (For‐ ssk.) Hayne	Fabaceae	Fruit/gum/resin/bark	Tree	Food/health benefit/fodder	Ayele and Negasa (2017), Tahir et al. (2023), Alemayehu et al. (2015), Gemedo‐Dalle et al. (2005), and Debela et al. (2012)
2	*Acokanthera scbimperi* (A. DC.) Schweinf	Apocynaceae	Fruit/leaf/whole parts	Tree	Food/health benefit/fodder	Regassa et al. (2015), Hankiso et al. (2023), Addis et al. (2013b), Nigatu et al. (2018), Wondimu et al. (2006), and Guzo et al. (2023)
3	*Balanites aegyptiaca* (L.) Del	Balanitaceae	Fruit/leaf/resin/flower/tendril	Tree	Food/health benefit/fodder	Kebede et al. (2017), Tahir et al. (2023), Demise (2020), Hankiso et al. (2023), Masresha et al. (2023), Tebkew et al. (2018), and Mekuanent et al. (2015)
4	*Bidens pachyloma*	Asteraceae	Leaf, young shoot	Herb	Food/feed/health benefit	Hankiso et al. (2023) and Regassa et al. (2015)
5	*Carissa spairanum* L.	Apocynaceae	Fruit/root/stem/leaf	Shrub	Food/health benefit/fodder	Kebede et al. (2017), Alemayehu et al. (2015), Yiblet and Adamu (2023), Tahir et al. (2023), Kidane et al. (2014), Tebkew (2015), and Guzo et al. (2023)
6	*Corchorus olitorius* L.	Malvaceae	Leaf	Herb	Food/health benefit/fodder	Balemie and Kebebew (2006), Masresha et al. (2023), Ayele and Negasa (2017), Addis et al. (2013b), Teklehaymanot and Giday (2010), and Tebkew et al. (2018)
7	*Cordia africana*	Boraginaceae	Fruit/leaf/stem/bud	Tree	Food/health benefit/fodder	Kebede et al. (2017), Yiblet and Adamu (2023), Abdella et al. (2023), Masresha et al. (2023), Tuasha et al. (2018), and Addis et al. (2013b)
8	*Cordia monica* Roxb.	Boraginaceae	Fruit/leaf/root	Shrub	Food/Health benefit/Fodder	Debela et al. (2012), Tahir et al. (2023), Addis et al. (2013b), Dechassa et al. (2012), Dejene et al. (2020), and Leta (2016)
9	*Dobera glabra* (Forssk.) Juss. ex Poir.	Salvadoraceae	Seed/fruit/leaf	Tree	Food/health benefit/fodder	Balemie and Kebebew (2006), Dechassa et al. (2012), Addis et al. (2013b), Teklehaymanot and Giday (2010), Debela et al. (2012), and Ketema et al. (2013)
10	*Dombeya aethiopica* Gilli	Malvaceae	Fruit/leaf/stem	Shrub	Food/health benefit/fodder	Tahir et al. (2023), Ketema et al. (2013), and Ayele and Negasa (2017)
11	*Embelia schimperi* Vatke	Myrsinaceae	Fruit	Shrub	Food/health benefit/fodder	Demise (2020), Yimer et al. (2023c), Tahir et al. (2023), Guzo et al. (2023), Regassa et al. (2015), and Alemneh (2020)
12	*Euclea racemosa*	Ebenaceae	Fruit/root	Shrub	Food/health benefit/fodder	Yiblet and Adamu (2023), Guzo et al. (2023), Alemayehu et al. (2015), Abera and Belay (2022), and Dechassa et al. (2012)
13	*Ficus sycomorus* L.	Moraceae	Fruit	Tree	Food/fodder/health benefit	Asfaw et al. (2022), Addis et al. (2013b), Addis et al. (2013b), Debela et al. (2012), Mekuanent et al. (2015), and Tebkew et al. (2018)
14	*Ficus thonningi* Blume.	Moraceae	Fruit	Tree	Food/health benefit/fodder	Ayele and Negasa (2017), Addis et al. (2013b), Dechassa et al. (2012), Tebkew et al. (2018), and Regassa et al. (2015)
15	*Ficus vasta* Forssk	Moraceae	Fruit	Tree	Food/health benefit/fodder	Addis et al. (2013b), Hankiso et al. (2023), Berihun and Molla (2017), Leta (2016), and Alemneh (2020)
16	*Flacourtia indica* (Burm.f.) Merr	Flacourtiaceae	Fruit	Shrub	Food/health benefit/fodder	Demise (2020), Hankiso et al. (2023), Tahir et al. (2023), Regassa et al. (2015), Assefa and Abebe (2011), and Ayele and Negasa (2017)
17	*Gardenia ternifolia* Schumach & Thonn.	Rubiaceae	Fruit/leaf/root	Shrub	Food/health benefit/fodder	Mekuanent et al. (2015), Tebkew et al. (2018), Getnet et al. (2015), Dejene et al. (2020), Nigatu et al. (2018), and Mekuanent et al. (2014)
18	*Grewia bicolar* Juss	Tiliaceae	Fruit/leaf	Shrub	Food/health benefit/fodder	Dechassa et al. (2012), Hassen (2021), Gemedo‐Dalle et al. (2005), Kebede et al. (2017), and Asfaw et al. (2022)
19	*Grewia ferruginea*	Malvaceae	Fruit/leaf/bark/	Shrub	Food, health benefit/fodder	Masresha et al. (2023), Alemayehu et al. (2015), Addis et al. (2013b), Dejene et al. (2020), and Asfaw et al. (2022)
20	*Grewia villosa* Will	Tiliaceae	Fruit/leaf/stem	Shrub	Food/health benefit/fodder	Kidane et al. (2014), Wondimu et al. (2006), Dejene et al. (2020), and Assefa and Abebe (2011)
21	*Hibiscus esculentus* L.	Malvaceae	Leaf	Herb	Food/health benefit/fodder	Tebkew et al. (2018), Mekuanent et al. (2015), and Mekuanent et al. (2014)
22	*Maytenus undata* (Thunb.) Blakelock	Celasteraceae	Fruit/bark	Shrub	Food/health benefit/fodder	Tahir et al. (2023) and Nigatu et al. (2018)
23	*Opuntia ficus‐indica*	Cactaceae	Fruit	Shrub	Food/health benefit/fodder	Seyoum et al. (2015), Asfaw and Tadesse (2001), Assefa and Abebe (2011), Abera and Belay (2022), Alemneh (2020), and Kebede et al. (2017)
24	*Papea capensis* Eckl. & Zeyh.	Sapindaceae	Fruit/bark	Tree	Food/health benefit/fodder	Wondimu et al. (2006), Demise (2020), and Tahir et al. (2023)
25	*Plumbago zeylanica* L.	Plumbaginaceae	Fruit/leaf	Herb	Food/health benefit/fodder	Getnet et al. (2015), Ketema et al. (2013), and Tebkew et al. (2018)
26	*Rhus natalensis*	Anacardiaceae	Fruit/leaf	Shrub	Food/health benefit/fodder	Yiblet and Adamu (2023), Alemayehu et al. (2015), Wondimu et al. (2006), and Leta (2016)
27	*Ricinus communis* L	Euphorbiaceae	Seed/root/leaf	Shrub	Food/health benefit/fodder	Demise (2020), Alemayehu et al. (2015), Ayele and Negasa (2017), and Nigatu et al. (2018)
28	*Rosa abyssincia*	Rosaceae	Fruit	Shrub	Food/fodder/health benefit	Demise (2020), Alemneh (2020), Yiblet and Adamu (2023), Abera and Belay (2022), Leta (2016), and Asfaw et al. (2022)
29	*Rumex nervosus*	Polygonaceae	Young stem/leaf/root	Shrub	Food/health benefit/fodder	Kebede et al. (2017), Demise (2020), Regassa et al. (2015), Dejene et al. (2020), and Abera and Belay (2022)
30	*Snowdenia polystachya*	Poaceae	Seed	Herb	Food/health benefit, fodder	Yiblet and Adamu (2023), Asfaw and Tadesse (2001), and Asfaw et al. (2022)
31	*Tamarindus indica*	Fabaceae	Fruit/flower	Tree	Food, feed, health benefit	Abdella et al. (2023), Masresha et al. (2023), Asfaw and Tadesse (2001), and Mekuanent et al. (2015)
32	*Terminalialaxiflora*	Combretaceae	Flower	Shrub	Food, feed, health benefit	Masresha et al. (2023) and Ayele and Negasa (2017)
33	*Vernonia amygdalina* Del	Asteraceae	Leaf/root/twigs	Shrub	Food/health benefit/fodder	Berihun and Molla (2017), Regassa et al. (2015), Alemneh (2020), and Nigatu et al. (2018)
34	*Ziziphus mucronata*	Rhamnaceae	Fruit	Tree	Food/health benefit/fodder	Kebede et al. (2017), Demise (2020), Alemayehu et al. (2015), Hassen (2021), Addis et al. (2013b), and Assefa and Abebe (2011)
35	*Ziziphus spina* Christi	Rhamnaceae	Fruit/leaf	Tree	Food/health benefit/fodder	Alemayehu et al. (2015), Getnet et al. (2015), Tahir et al. (2023), Hassen (2021), and Kebede et al. (2017)

### 3.4 Cultivation of WEPs

The cultivation of WEPs is gaining attention as a sustainable alternative to conventional agriculture. These plants, often resilient to extreme weather, pests, and diseases, require minimal inputs, making them valuable for ecological conservation and food security (FAO 2021). Unlike domesticated crops, wild edibles are naturally adapted to local environments, reducing the need for chemical fertilizers and pesticides while enhancing soil health and biodiversity (Harlan 1992).

Propagation of WEPs can be achieved through various methods, including seed sowing, cuttings, and transplanting. Many species, such as wild garlic ( *Allium ursinum* ) and purslane ( *Portulaca oleracea* ), readily reseed themselves, ensuring long‐term sustainability. Soil preparation should mimic the plant's native habitat, incorporating organic matter like compost or leaf mulch to maintain fertility. Minimal irrigation is required, as many wild plants are drought‐tolerant, reducing water usage in agricultural systems (Chivenge et al. 2015).

From an ecological perspective, integrating WEPs into agroforestry and permaculture systems fosters biodiversity and soil conservation. These plants support pollinators and wildlife while preventing soil erosion, making them essential for regenerative agriculture (Altieri 1999). Additionally, their cultivation contributes to food sovereignty by providing nutrient‐rich, locally adapted alternatives to conventional crops, which is particularly crucial in regions affected by climate change and food insecurity (Padulosi et al. 2013).

Sustainable harvesting practices, such as rotational foraging and seed‐saving, ensure that WEP populations remain abundant for future generations. Moreover, commercializing cultivated wild edibles presents economic opportunities for small‐scale farmers and indigenous communities (Heywood 2011). As awareness of wild plants' nutritional and ecological benefits grows, their integration into mainstream agriculture can contribute to a more resilient and sustainable food system (Pardo‐De‐Santayana et al. 2005).

### 3.5 Mode of Consumption and Processing of WEPs

The consumption of WEPs is diverse, ranging from raw consumption to cooking, fermentation, and preservation, each offering distinct nutritional and culinary benefits. Many wild greens, such as dandelion ( *Taraxacum officinale* ), wild mustard ( *Sinapis arvensis* ), and lamb's quarters ( *Chenopodium album* ), are often eaten fresh in salads, sandwiches or as garnishes, allowing them to retain essential nutrients like vitamin C, fiber, and minerals (Grivetti and Ogle 2000). Wild fruits, such as blackberries ( *Rubus fruticosus* ) and elderberries ( *Sambucus nigra* ), are frequently consumed fresh or incorporated into smoothies, fruit salads, and desserts, providing essential antioxidants and vitamins that support immune function. Similarly, wild roots and tubers, including wild yam (*Dioscorea* spp.) and Jerusalem artichoke ( *Helianthus tuberosus* ), can be eaten raw but are often cooked to enhance their digestibility and flavor. Wild garlic ( *Allium ursinum* ), known for its antibacterial properties, is used raw and cooked in various dishes (Pérez‐Ovando 2017).

Cooking is a common method used to improve the taste and digestibility of wild plants. Boiling or steaming wild greens such as nettles ( *Urtica dioica* ) and fiddlehead ferns ( *Matteuccia struthiopteris* ) helps neutralize natural toxins, making them safer to eat and suitable for inclusion in soups, stews, or side dishes (Kadioglu et al. 2024). Stir‐frying or sautéing wild herbs like wild thyme ( *Thymus vulgaris* ) and wild onions ( *Allium cepa* ) enhance their flavor. At the same time, soups and stews benefit from the addition of wild mushrooms, such as chanterelles (*Cantharellus* spp.) and wild carrots ( *Daucus carota* ), which contribute depth and richness to the dish (Bruni et al. 2019). Additionally, fermentation is a traditional preservation technique that extends the shelf life of wild plants and boosts their probiotic content. Fermented wild cabbage ( *Brassica oleracea* ) and wild garlic are known for their gut health benefits and enhanced nutritional value (Yan et al. 2023).

Various preservation methods, including drying, canning, and freezing, enable wild plants to be stored for extended periods and enjoyed beyond their growing season. Dried wild fruits and herbs, such as elderberries and chamomile ( *Matricaria chamomilla* ), are commonly used in teas, jams, and flavoring ingredients (Al‐Juhaimi et al. 2018). Canning wild vegetables like wild carrots and beans ensures their availability year‐round for culinary use (Pérez‐Ovando 2017). Many WEPs are also valued for their medicinal benefits, such as dandelion tea, which has been traditionally consumed for its detoxifying and liver‐supporting properties (Grivetti and Ogle 2000). Overall, the inclusion of WEPs in the diet offers a sustainable, nutrient‐rich alternative to conventional foods, providing both health benefits and diverse culinary possibilities.

### 3.6 Plant Growth Forms of WEPs in Ethiopia

**FIGURE 4:**
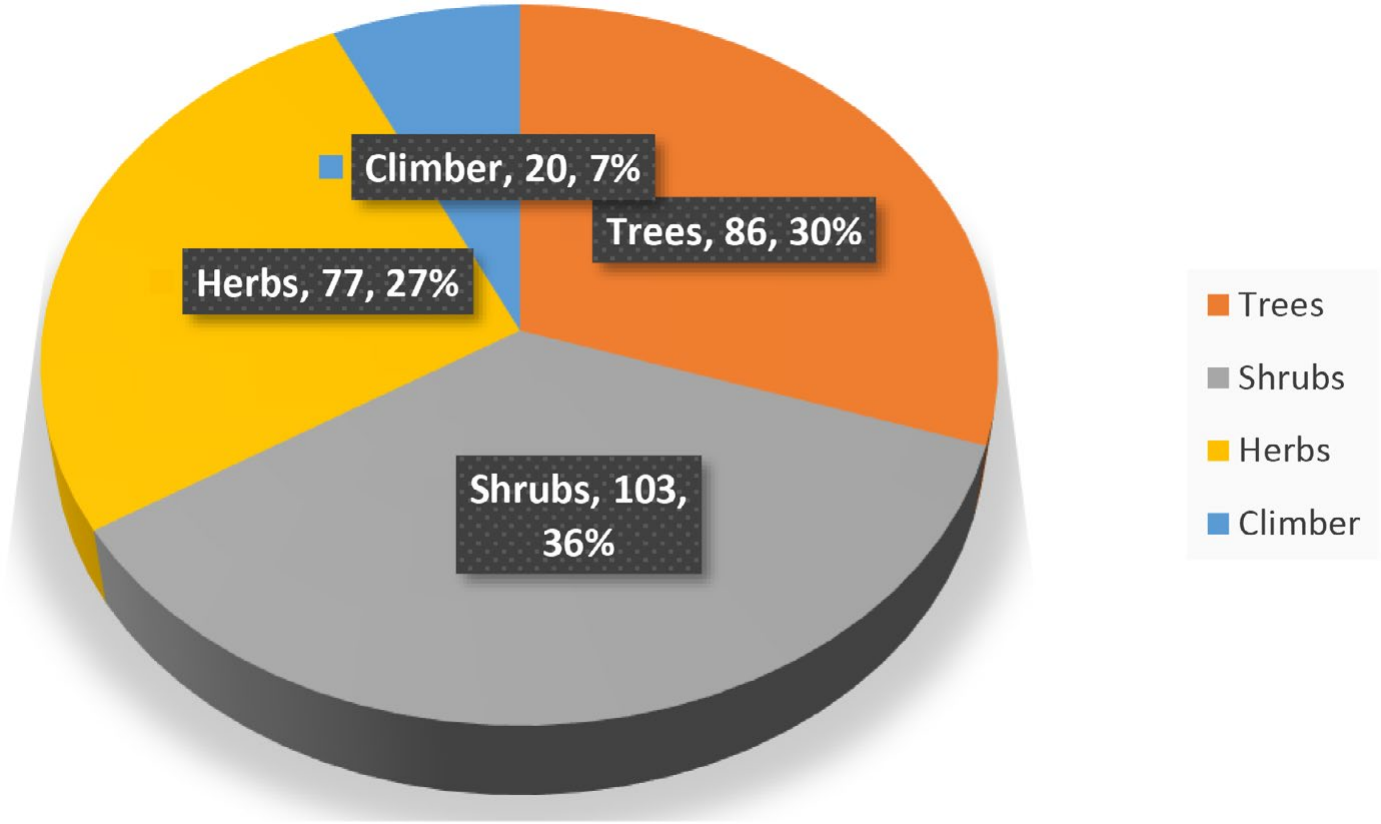
Proportion of plant growth forms of WEPs in Ethiopia.

The reports indicated that shrubs (*n* = 103) account for relatively large growth forms coverage of WEPs, followed by trees (*n* = 86), herbs (*n* = 77), and climbers (*n* = 20). The information on the proportion of plant growth forms of WEPs in Ethiopia is presented in Figure 4.

The nutritional composition of some WEPs studied in Ethiopia by different researchers is indicated in Table 3.

**TABLE 3:** Nutritional and mineral contents of some analyzed wild edible plants.

WEPs	Country	Moisture (%)	Energy (Kcal/100 g)	Protein (g/100 g)	Fat (g/100 g)	CHO (g/100 g)	Ca (mg/100 g)	Fe (mg/100 g)	Zn (g/100 g)	References
*Aframomum daniellii*	Cameroon	10.4 (db)	—	8.5	23.1	11.9	62.0	27.6	6.7	Abdou Bouba et al. (2012)
*Allium astrosanguineum*	Pakistan	5.33 (db)	276.16	17.53	5.98	33.58	5.47	5.84	0.27	Ullah et al. (2017)
*Amaranthus gangeticus*	Bangladesh	—	—	—	—	—	2596	47.3	9.54	Hels et al. (2004)
*Amaranthus gangeticus*	Bangladesh	—	—	—	—	—	1521	19.7	6.93	Hels et al. (2004)
*Amaranthus graecizans*	Ethiopia	—	141.99	—	—	—	2065	91.29	3.81	Aragaw et al. (2021)
*Amaranthus hybridus*	Côte D'ivoire	72.98 (wb)	305.19	13.25	2.15	58.21	1271.2	—	—	Patricia et al. (2014)
*Amaranthus thunbergii*	Pakistan	3.94 (db)	278.41	17.19	6.07	34.70	11.63	3.5	0.69	Ullah et al. (2017)
*Andasonia digitata*	Côte D'ivoire	77.63 (wb)	267.03	18.08	2.18	56.23	3532.3	38.39	—	Patricia et al. (2014)
*Balanites aegeyptica*	Ethiopia	11.22 (dwb)	266.5	7.78	0.54	57.67	152.21	31.21	1.0	Mokria et al. (2022)
*Balanites aegyptiaca*	Ethiopia	63.5 (wb)	59	10.5	0.9	14.9	908	4.90	0.4	Getachew et al. (2013)
*Brassica campestris*	Bangladesh	—	—	—	—	—	2440	49.4	12.6	Hels et al. (2004)
*Capsicum frutescens*	Cameroon	9.3 (db)	—	9.4	11.1	7.0	173.0	6.4	3.7	Abdou Bouba et al. (2012)
*Carisa spinarum*	Ethiopia	—	252.3	—	—	—	130	4.4	1.33	Aragaw et al. (2021)
*Carissa Spinarum*	Ethiopia	84.2	62	0.5	1.3	13.6	11	1.31	—	Gebru et al. (2019)
*Carraluma edulis*	Pakistan	4.11 (db)	277.7	17.34	6.85	32.83	5.39	2.31	0.38	Ullah et al. (2017)
*Ceiba patendra*	Côte D'ivoire	70.45 (wb)	142.61	15.20	1.39	26.30	4610.0	80.0	—	Patricia et al. (2014)
*Chenopodium murale*	Pakistan	83.33 (wb)	69.24	4.89	1.00	10.53	1125.3	24.96	23.5	Khan et al. (2016)
*Cleome gynandra*	Ethiopia	7.1 (dwb)	276.0	20.1	3.3	41.4	594.8	21.7	5.5	Yimer et al. (2023a)
*Cordia africana*	Ethiopia	11.21 (dwb)	277.97	8.1	0.69	60.26	96.55	28.51	0.95	Mokria et al. (2022)
*Dioscorea cayenensis*	Ethiopia	—	182.96	—	—	—	1225	46.78	3.83	Aragaw et al. (2021)
*Dioscorea praehensilis*	Ethiopia	5.2 (dwb)	354.1	4.0	0.7	83.0	3.7	3.4	5.9	Yimer et al. (2023a)
*Dovyalis Abyssinica*	Ethiopia	9.83 (dwb)	342.63	3.01	1.46	78.27	120.18	2.09	0.62	Keyata et al. (2020)
*Eruca sativa*	Pakistan	88.00 (wb)	28.04	2.88	0.40	8.23	1102.2	30.86	28.43	Khan et al. (2016)
*Fagara leprieuri*	Cameroon	9.5 (db)	—	6.4	32.1	3.4	182.0	18.3	1.5	Abdou Bouba et al. (2012)
*Ficus mucuso*	Ethiopia	10.64 (dwb)	337.71	5.11	3.31	71.87	190.18	20.96	0.62	Keyata et al. (2020)
*Goldbachia laevigata*	Pakistan	76.96 (wb)	91.69	3.60	0.89	17.89	1102.2	36.38	34.1	Khan et al. (2016)
*Hibiscus sabdariffa*	Côte D'ivoire	86.05 (wb)	275.71	14.47	4.75	56.21	1791.7	30.87	—	Patricia et al. (2014)
*Indica Gardenia Erubescens*	Ethiopia	10.01 (dwb)	341.99	4.22	1.4	77.88	98.89	15.04	6.23	Keyata et al. (2020)
*Malcolmia africana*	Pakistan	87.00 (wb)	32.52	2.92	1.52	7.79	920.0	38.00	30.0	Khan et al. (2016)
*Malva neglecta*	Pakistan	83.00 (wb)	57.30	3.88	0.58	10.14	1060.0	110.0	30.0	Khan et al. (2016)
*Malva neglecta*	Turkey	85.08 (wb)	—	16.9	—	—	613.22	—	—	Kibar and Kibar (2017)
*Medicago polymorpha*	Pakistan	86.75 (wb)	44.67	2.66	0.35	9.72	933.6	106.0	22.5	Khan et al. (2016)
*Melilotus officinalis*	Pakistan	70.50 (wb)	140.6	5.28	0.93	14.78	1060.0	60.0	20.0	Khan et al. (2016)
*Mimusops Kummel*	Ethiopia	13.1 (dwb)	—	2.2	1.6	—	80.5	—	2.95	Fentahun and Hager (2009)
*Monodora myristica*	Cameroon	7.7 (db)	—	13.6	53.4	0.4	375.0	36.7	1.4	Abdou Bouba et al. (2012)
*Nasturtium officinale*	Pakistan	87.50 (wb)	32.29	2.85	0.81	7.40	870.0	25.0	40.0	Khan et al. (2016)
*Piper guineense*	Cameroon	9.2 (db)	—	8.5	20.3	9.6	466.0	21.8	5.2	Abdou Bouba et al. (2012)
*Piper umbellatum*	Cameroon	8.7 (db)	—	10.4	6.0	9.0	845.0	97.8	2.4	Abdou Bouba et al. (2012)
*Polygonum cognatum*	Turkey	83.43 (wb)	—	17.4	—	—	289.43	—	—	Kibar and Kibar (2017)
*Portulaca oleracea*	Ethiopia	—	97.2	—	—	—	785	44.51	4.33	Aragaw et al. (2021)
*Portulacca olaracea*	Pakistan	4.71 (db)	240.3	24.2	6.46	18.00	5.30	4.12	0.07	Ullah et al. (2017)
*Rubus apetalus*	Ethiopia	—	151	—	—	—	150	18.48	6.51	Aragaw et al. (2021)
*Rumex patientia*	Pakistan	3.20 (db)	299.6	24.4	6.08	32.22	5.31	3.03	0.06	Ullah et al. (2017)
*Rumex vesicarius*	Saudi Arabia	—	—	18.6	3.4	—	2840	36.2	5.4	Alfawaz (2006)
*Solanum melongena*	Bangladesh	—	—	—	—	—	126	3.53	2.13	Hels et al. (2004)
*Solanum nigrum*	Ethiopia	6.0	275.0	21.7	4.0	38.1	241.1	26.9	3.7	Yimer et al. (2023a)
*Strychnos cocculoides*	Tanzania	79.5 (wb)	143.0	0.11	0.003	35.64	38.71	10.27	0.01	Mapunda and Mligo (2019)
*Sysygium guineense*	Ethiopia	—	244.5	—	—	—	65	24.9	1.38	Aragaw et al. (2021)
*Syzygium guineense*	Tanzania	85.9 (wb)	66.24	0.23	0.001	16.33	44.42	1.76	0.04	Mapunda and Mligo (2019)
*Tamarindus*	Ethiopia	41.3 (wb)	—	2.4	1.1	53.0	—	2.08	—	Fentahun and Hager (2009)
*Trachystemon orientalis*	Turkey	87.93 (wb)	—	18.9	—	—	548.69	—	—	Kibar and Kibar (2017)
*Trilepisium madagascariense*	Ethiopia	7.9 (dwb)	371.1	6.3	6.1	72.6	57.4	0.8	2.4	Yimer et al. (2023a)
*Uapaca kirkiana*	Tanzania	82.3 (wb)	227.8	0.94	0.001	56.01	58.96	5.65	0.01	Mapunda and Mligo (2019)
*Vigna membranacea*	Ethiopia	5.9 (dwb)	286.6	11.8	4.3	50.3	322.8	38.5	3.9	Yimer et al. (2023a)
*Vigna unguiculata*	Côte D'ivoire	80.04 (wb)	248.4	21.96	4.23	44.64	3357.3	45.80	—	Patricia et al. (2014)
*Ximenia caffra*	Tanzania	84.0 (wb)	100.1	0.72	0.0001	24.31	23.18	20.39	0.02	Mapunda and Mligo (2019)
*Ziziphus spina‐christi*	Ethiopia	7.6 (dwb) (wb)	79	3.2–4.8	0.9–1.2	80.7	140	—	—	Fentahun and Hager (2009) and Saied et al. (2008)
*Ziziphus spania‐chrsti*	Ethiopia	8.23 (dwb)	325.2	5.3	1.3	73.06	190.66	14.7	0.35	Mokria et al. (2022)
*Ziziphus spina‐chris*	Ethiopia	13.1 (dwb)	316.1	5.31	1.65	70.41	67.17	29.13	8.34	Keyata et al. (2020)

Some WEP distributed in Ethiopia are reported to contain phytochemicals and essential nutrients, as stated in Table 4.

**TABLE 4:** Phytochemical contents of some wild edible plants.

WEPs	Country	Phytochemical contents				References
		Vitamin C (mg/100 g)	Beta‐carotene (mg/100 g)	Total phenol (mg GAE/100 g)	Total flavonoid (mg CE/100 g)	
*Adansonia digitata*	Burkina Faso	—	—	3518.33	31.70	Lamien‐Meda et al. (2008)
*Aegle marmelos*	Thailand	—	—	8168	2168	Kubola et al. (2011)
*Amaranthus graecizans*	Ethiopia	180.7	—	—	—	Aragaw et al. (2021)
*Amaranthus hybridus*	Côte D'ivoire	55.00	—	238.67	27.00	Patricia et al. (2014)
*Amaranthus hybridus leaf*	Ethiopia	2.36	—	—	—	Adamu et al. (2022)
*Amorphophallus abyssinicus*	Ethiopia	—	—	110.70	5.21	Tsehay et al. (2023)
*Andasonia digitata*	Côte D'ivoire	70.00	—	135.21	16.40	Patricia et al. (2014)
*Balanites aegyptiaca*	Ethiopia	2.32	—	47.84	21.54	Tafesse (2018)
*Carisa spinarum*	Ethiopia	256.55	—	—	—	Aragaw et al. (2021)
*Carissa carandas*	Thailand	—	—	180	1469	Kubola et al. (2011)
*Ceiba patendra*	Côte D'ivoire	40.00	—	293.08	16.55	Patricia et al. (2014)
*Chenopodium murale*	Pakistan	—	—	—	25.60	Khan et al. (2016)
*Chrysophyllum cainito*	Thailand	—	—	1788	1117	Kubola et al. (2011)
*Cleome gynandra*	Ethiopia	23.40	28.67	29.03	11.25	Yimer et al. (2023b)
*Coccinia grandis*	Thailand	—	—	690	371	Kubola et al. (2011)
*Dovyalis abyssinica*	Ethiopia	—	—	191.36	91.51	Jiru et al. (2023)
*Dioscorea cayenensis*	Ethiopia	259.33	—	—	—	Aragaw et al. (2021)
*Dioscorea praehensilis*	Ethiopia	10.0	11.81	0.25	0.85	Yimer et al. (2023b)
*Dovyalis abyssinica*	Ethiopia	112.9	—	80.24	29.08	Tafesse (2018)
*Eruca sativa*	Pakistan	—	—	—	25.50	Khan et al. (2016)
*Erucastrum abyssinicum*	Ethiopia	70.42	—	—	—	Adamu et al. (2022)
*Erucastrum arabicum*	Ethiopia	23.31	—	—	—	Adamu et al. (2022)
*Ficus mucuso*	Ethiopia	3.00	—	22.13	38.10	Tafesse (2018)
*Ficus mucuso*	Ethiopia	—	—	191.61	88.10	Jiru et al. (2023)
*Ficus sur*	Burkina Faso	—	—	247	19.78	Ramde‐Tiendrebeogo et al. (2012)
*Gardenia erubescens*	Ethiopia	—	—	230.76	112.85	Jiru et al. (2023)
*Goldbachia laevigata*	Pakistan	—	—	—	60.75	Khan et al. (2016)
*Haplocarpha schimperi*	Ethiopia	12.77	—	—	—	Adamu et al. (2022)
*Hibiscus sabdariffa*	Côte D'ivoire	30.00	—	251.12	27.58	Patricia et al. (2014)
*Lannea microcarpa*	Burkina Faso	—	—	240.58	23.35	Lamien‐Meda et al. (2008)
*Malcolmia africana*	Pakistan	—	—	—	45.90	Khan et al. (2016)
*Malva neglecta*	Pakistan	—	—	—	25.60	Khan et al. (2016)
*Medicago polymorpha*	Pakistan	—	—	—	85.75	Khan et al. (2016)
*Melilotus officinalis*	Pakistan	—	—	—	20.34	(Khan et al. 2016)
*Mimusops kummel*	Ethiopia	30.34	—	46.7	17.32	Tafesse (2018)
*Moringa stenopetala*	Ethiopia	—	—	3900.0	1100	Dessalegn and Rupasinghe (2021)
*Nasturtium officinale*	Pakistan	—	—	—	27.35	Khan et al. (2016)
*Portulaca oleracea*	Ethiopia	191.02	—	—	—	Aragaw et al. (2021)
*Pouteria campechiana*	Thailand	—	—	500	458	Kubola et al. (2011)
*Rubus apetalus*	Ethiopia	294.19	—	—	—	Aragaw et al. (2021)
*Rumex nervosus*	Ethiopia	2.16	—	—	—	Adamu et al. (2022)
*Rumex vesicarius*	Saudi Arabia	253	—	—	—	Kibar and Kibar (2017)
*Solanum nigrum*	Ethiopia	35.90	19.15	35.73	8.65	Yimer et al. (2023b)
*Spondias pinnata*	Thailand	—	—	4678	539	Kubola et al. (2011)
*Strychnos cocculoides*	Tanzania	53.91	—	255.38	55.97	Mapunda and Mligo (2019)
*Sysygium guineense*	Ethiopia	330.72	—	—	—	Aragaw et al. (2021)
*Syzygium guineense*	Tanzania	121.67	—	137.37	45.24	Mapunda and Mligo (2019)
*Tamarindus*	Ethiopia	11.3	—	—	—	Fentahun and Hager (2009)
*Trilepisium madagascariense*	Ethiopia	26.07	24.92	22.97	2.07	Yimer et al. (2023b)
*Uapaca kirkiana*	Tanzania	93.03	—	492.31	74.11	Mapunda and Mligo (2019)
*Urtica simensis*	Ethiopia	12.18	—	—	—	Adamu et al. (2022)
*Vigna membranacea*	Ethiopia	45.0	34.49	27.23	5.35	Yimer et al. (2023b)
*Vigna unguiculata*	Côte D'ivoire	60.00	—	136.03	15.00	Patricia et al. (2014)
*Ximenia americana*	Burkina Faso	—	—	2230.0	30.95	Lamien‐Meda et al. (2008)
*Ximenia caffra*	Tanzania	358.82	—	1915.88	178.46	Mapunda and Mligo (2019)
*Ziziphus spina‐christi*	Ethiopia	30.0	—	—	—	Fentahun and Hager (2009) and Saied et al. (2008)
*Ziziphus mauritiana*	Burkina Faso	—	—	2352.50	56.88	Lamien‐Meda et al. (2008)
*Ziziphus spina‐christi*	Ethiopia	29.43	—	35.84	48.34	Tafesse (2018)
*Ziziphus spina‐christi*	Ethiopia	—	—	108.32	79.70	Jiru et al. (2023)

### 3.7 Health Benefits of WEPs and Mechanisms of Action

Many WEPs, such as vegetables and fruits, are rich in phytochemicals like vitamins (β‐carotene, vitamins C and E) and polyphenols (flavonoids, tannins, catechins), which exhibit antioxidant properties (Yimer et al. 2023b; Wong et al. 2006; Kwinana‐Mandindi 2015; Tsehay et al. 2023; Jiru et al. 2023; Deng et al. 2013). Phytochemicals are naturally occurring, biologically active compounds in plants that offer health benefits beyond providing essential nutrients. These compounds, considered secondary metabolites, have a range of biological activities, including antioxidant effects, anti‐cancer properties, antimicrobial effects, regulation of enzyme detoxification, modulation of the immune system, reduced platelet aggregation, hormone metabolism, and more (Saxena et al. 2013).

Oxygen, essential for sustaining life, can also harm the human body under certain conditions. Plants produce it through photosynthesis, which plays a crucial role in animal aerobic respiration. During cellular growth, oxygen use leads to the formation of free radicals, including reactive oxygen species (ROS) and reactive nitrogen species (RNS), as well as specific radicals like superoxide anion (O_2_^−2^) and hydroxyl radicals (OH^−^). Non‐radical molecules such as hydrogen peroxide (H_2_O_2_) and singlet oxygen (^1^O_2_) are also generated. These byproducts arise naturally through respiration and immune system activities (Gülçin 2006; Farombi and Fakoya 2005; Gülçin et al. 2002). ROS are continuously formed during normal physiological processes and can cause lipid peroxidation in cell membranes, accumulating lipid peroxides. While ROS are essential for cellular function at moderate levels, excessive amounts can harm crucial biomolecules, including nucleic acids, proteins, lipids, polyunsaturated fatty acids, and carbohydrates. This oxidative stress may result in DNA damage and potential mutations. If the body's defense mechanisms fail to neutralize excess ROS, they can initiate chain reactions that further damage proteins, lipids, and nucleic acids, contributing to the development of various diseases (Gülçın et al. 2003; Gutteridge 1990).

Free radicals can damage cell membranes through various mechanisms. First, they may form covalent bonds with membrane components, altering receptor activity. Second, they can oxidize thiol (‐SH) groups in membrane proteins, disrupting essential transport functions. Third, free radicals can initiate lipid peroxidation, primarily targeting polyunsaturated fatty acids (PUFAs). Key targets of free radical damage include proteins, unsaturated fatty acids, lipoproteins, and DNA components like carbohydrates, with unsaturated fatty acids being the most vulnerable (Winarsi 2007). The chain reactions triggered by free radicals generate additional radicals, leading to extensive cellular and tissue damage, which may contribute to autoimmune diseases, degenerative conditions, and cancer. Elevated free radical levels in the body are often reflected by increased plasma malondialdehyde (MDA) concentrations and decreased antioxidant enzyme activity (Winarsi 2007).

WEPs contain numerous phytochemicals, such as vitamins C and E, carotenoids, chlorophyll derivatives, alkaloids, flavonoids, phenolic acids, and other phenols known for their antioxidant properties. Antioxidants help neutralize free radicals and prevent the oxidation of essential molecules. They achieve this by oxidizing themselves, interrupting the free radical damage chain reaction, and binding catalytic metals to inhibit further oxidative processes (Sies 1996).

Certain phytochemicals modulate inflammatory pathways, thereby reducing inflammation. For example, flavonoids in onions and berries inhibit enzymes like cyclooxygenase and lipoxygenase, decreasing the production of pro‐inflammatory mediators. This action can alleviate symptoms of inflammatory conditions and lower the risk of chronic diseases (Kussmann et al. 2023).

Certain phytochemicals exhibit strong antibacterial properties by disrupting bacterial cell membranes, inhibiting essential enzymes, and interfering with DNA replication. They may also compromise the bacterial cytoplasmic membrane, leading to leakage of cellular contents, or inhibit key enzymes necessary for cell wall synthesis and energy production. For instance, allicin, a garlic compound, effectively combats Gram‐positive and Gram‐negative bacteria by targeting bacterial enzymes, thereby weakening cell membrane integrity (Okafor et al. 2025).

Phytochemicals exhibit potential anticancer properties by inducing apoptosis, inhibiting cell proliferation, and preventing angiogenesis. These compounds disrupt cancer cell signaling pathways, making them valuable in cancer research and therapy. For example, curcumin and epigallocatechin gallate promote cancer cell apoptosis by altering mitochondrial membrane potential, triggering cytochrome c release, and activating caspases. Additionally, they suppress anti‐apoptotic proteins like Bcl‐2 while increasing pro‐apoptotic molecules such as Bax, further promoting cancer cell death (Batool et al. 2025).

Phytochemicals can influence metabolic processes. For example, ginger contains compounds like gingerol and shogaol that stimulate digestive enzymes, enhancing gastrointestinal motility and alleviating digestive discomfort. Additionally, these compounds have been shown to improve blood sugar levels and circulation (Espejo 2016).

## 4 Discussion

The present study reviews the literature on WEPs and their use as a food, feed, and pharmaceutical source in Ethiopia. Ethiopia has a vast diversity of WEPs, with 74 plant families and 679 species reportedly utilized for food, animal feed, and pharmaceuticals. These plants continue to play a crucial role in the daily lives of local communities across different regions, serving as essential resources for nutrition, traditional medicine, and food security.

Globally, the disparity between plant diversity and human utilization is striking. Out of approximately 350,000 known plant species, around 80,000 are considered edible to humans. However, only 150 species are actively cultivated for food production (Léder 2009). This means less than 0.05% of the world's plant species are systematically grown for human consumption, despite the vast pool of available edible plants.

Ethiopia's use of 679 WEP species stands out as an example of how traditional knowledge helps communities tap into a broader range of plant resources beyond conventional crops. In contrast, global agriculture focuses heavily on a few staple crops such as wheat, rice, and maize. Many WEPs in Ethiopia provide essential nutrients, especially during food scarcity. These plants also offer resilience to climate change, as they often thrive in harsh environments where cultivated crops may fail. The stark contrast between the global underutilization of plant diversity and Ethiopia's relatively high reliance on WEPs highlights the untapped potential of these species in addressing global food security, dietary diversity, and sustainability. Recognizing and integrating WEPs into mainstream agricultural systems could help reduce dependence on a narrow range of staple crops and promote biodiversity conservation.

The reviewed literature indicated that communities in different regions of the country use several WEPs for food, feed, and/or health benefits. This usage suggests that the local community's longstanding and rich traditions and knowledge could be used as primary data sources. Thus, further investigation is needed to explore the communities' traditional knowledge of using WEPs for various purposes. Despite the rich potential of WEPs in Ethiopia, studies describing ethnobotanical, nutritional, phytochemical contents, antioxidant activities, and toxicity of some WEPs are minimal (Kibar and Kibar 2017). Thus, implementing well‐designed, comprehensive, and scientifically sound investigations is vital to efficiently utilize locally available resources to solve challenges concerning nutrition deficiencies and access to safe and effective herbal health benefits.

According to the reviewed literature, communities in different regions of Ethiopia use WEPs mainly for food/feed and health benefits. However, if various features of WEPs are adequately identified, they could also be utilized for soil conservation and environmental protection, home and feed for wildlife, construction of houses and fences, firewood, charcoal, beekeeping, furniture, and paper industries. Therefore, identifying WEPs that could be used for the abovementioned purposes and cultivating them on large scales at appropriate sites needs due attention and collaboration of various stakeholders.

The results indicate that WEP families (Fabaceae, Moraceae, and Malvaceae) and species (*Syzygium guineense, Cordia Africana, Carissa spinarum*, and *Ficus sur*) are the most commonly consumed WEPs by different communities in Ethiopia (Addis et al. 2013a; Berihun and Molla 2017; Hankiso et al. 2023; Amente 2017). This established cultural importance and traditional use for food and health benefits indicates that the aforementioned families and species of WEPs are well‐known and traditionally proven to be used for food and health benefits. Thus, it suggests that these families and species should be prioritized in further investigations focusing on exploring the nutrients and phytochemicals.

The fruits and leaves of *Syzygium* species, which are one of the commonly consumed WEP species in Ethiopia, reportedly claim to have essential nutrients and phytochemicals and thus are widely consumed for food and feed in different parts of the world (Uddin et al. 2022; Batista et al. 2017; Low et al. 2022). Phytochemicals such as phenolic acids, flavonoids, tannins, and dietary fiber promote health. Previous studies indicate that *Syzygium* species demonstrate antioxidant, antihyperglycemic, anti‐inflammatory, hepatoprotective, and antibacterial activities (Gibbert et al. 2021; Konstantinidi and Koutelidakis 2019; Tamiello et al. 2018; Sobeh et al. 2018; Nunes et al. 2016; Singh et al. 2016) and serve as a source of health benefits for the treatment of different diseases globally (Uddin et al. 2022; Cock and Cheesman 2018; de Araújo et al. 2024). Therefore, *Syzygium* species are vital sources of essential nutrients and phytochemicals that must be well investigated and efficiently utilized for various purposes.

*Cordia africana* Lam is a tree that produces small fruits widely eaten in Ethiopia and other parts of Africa (Balemie and Kebebew, 2006; Addis et al. 2005; Alemayehu et al. 2016). The fruit of *Cordia Africana* is one medicinal plant having essential phytochemicals such as total phenols, flavonoids, and tannins, which play a role in antioxidant activities in scavenging potent‐free radical activity and antimicrobial activity, and hence keep the health of humans (Ganesan et al. 2015; Salaudeen et al. 2022; Alhadi et al. 2015; Sabry et al. 2022). It also contains better nutritional contents of essential macronutrients (protein, carbohydrate, and dietary fiber) and micronutrients (minerals) that are crucial for growth, development, and preventing lifestyle diseases (Debela et al. 2017; Mokria et al. 2022; Tewolde‐Berhan et al. 2015). Due to its high nutritional content, phytochemicals, antioxidants, and antimicrobial activity, it can be used throughout the country for food, feed, and health benefits.

The fruits, leaves, and roots of *Carissa spinarum* are consumed globally for nutritional and health importance. It is rich in macronutrients and micronutrients essential for the proper functioning of the human body (Siyum and Meresa 2021; Jayakumar and Muthuraman 2018). Due to the possession of essential phytochemicals in *Carissa* species, it plays a role in antioxidant, anticancer, antimicrobial, anticonvulsant, cardioprotective, antipyretic, analgesic, wound healing, antiarthritic, adaptogenic, anti‐inflammatory, and antidiabetic activities, thus validating its use in indigenous health benefit systems (Dhatwalia et al. 2021; Wangteeraprasert et al. 2012; Ansari and Patil 2018).

The fruits of *Ficus sur* are widely distributed and consumed in Africa. It contains high carbohydrate content and excess essential micronutrients, especially minerals like calcium, iron, magnesium, zinc, manganese, copper, and selenium (Ogunlaja et al. 2017). It is also characterized by high contents of phytochemicals such as phenols and flavonoids, which have antioxidant activity in scavenging free radicals to prevent some lifestyle diseases in different parts of the world. It also has essential phytochemicals that initiate antioxidant and antimicrobial activities to be applied in pharmaceutical industries (Ansari and Patil 2018; Saloufou et al. 2018; Ogunlaja et al. 2022; Sieniawska et al. 2022). These findings reflect that the reported families and species are widely accepted across different communities in Ethiopia. Though the aforementioned families and species are widely consumed for various purposes, detailed scientific investigations supporting traditional knowledge have not been conducted. Thus, further studies are needed to explore the composition and safety of nutrients and active compounds.

WEPs are highly nutritious and serve as valuable sources of vitamins and micronutrients (Feyssa et al. 2011b). They can potentially alleviate micronutrient deficiencies, particularly in rural and Indigenous communities (Powell et al. 2015; Flyman and Afolayan 2006). Ensuring food and nutrition security requires incorporating diverse food sources, including wild plants. Many of these plants are rich in key nutrients such as protein, vitamin B_2_, and vitamin C, making them suitable alternatives to conventional vegetables (Fentahun and Hager 2009). Research indicates that wild foods often provide higher concentrations of vitamins, minerals, and other nutrients than cultivated crops. Studies on certain wild plants suggest their nutrient composition is comparable to or exceeds that of domesticated varieties (Kabuye 1997). Various plant parts, including fruits, leaves, stems, seeds, and roots, contribute essential nutrients to human diets (Dirres 2016). Although research on the nutritional composition of WEPs in Ethiopia is still limited, existing studies highlight their richness in vital nutrients. This gap emphasizes the need for further research on WEPs in Ethiopia, where these underutilized food sources could significantly enhance food security.

Since food security and access to essential health benefits are considerable challenges in low‐income countries, the reported 35 species of WEPs with edible parts serving as food/feed and health benefits are potential resources in alleviating such challenges. However, scientifically identifying WEP species that are safe and rich in nutrients and active compound composition is essential.

WEPs provide essential nutrients and improve food security, particularly in developing countries (Hunter et al. 2019). Rich in key nutrients, especially wild fruits, these plants help combat malnutrition, particularly among children (Fentahun and Hager 2009). They are abundant sources of essential vitamins and minerals, which help alleviate micronutrient deficiencies in rural and indigenous communities (Flyman and Afolayan 2006; Powell et al. 2015). Given their high nutritional content, promoting WEPs as an alternative food source is crucial for enhancing food and nutrition security. These plants offer significant protein, vitamin B_2_, and vitamin C, making them a valuable alternative to conventional fruits and vegetables (Fentahun and Hager 2009). Furthermore, many wild edible species contain high levels of vitamins, soluble fiber, and phytochemicals like ascorbic acid (Barros et al. 2010) while also being rich in essential micronutrients (Sánchez‐Mata et al. 2012; Martins et al. 2011).

The systematic review presents detailed nutritional and mineral composition data for a wide range of WEP across various countries, including Ethiopia, Pakistan, Cameroon, Côte d'Ivoire, Tanzania, Turkey, and Bangladesh. It highlights significant interspecies and interregional variation in key nutritional components such as moisture content, energy (kcal/100 g), protein, fat, carbohydrates (CHO), calcium (Ca), iron (Fe), and zinc (Zn).

One notable trend is the diversity in moisture content, which is reported either on a dry basis (db) or wet basis (wb), ranging from as low as around 3%, wb in *Rumex patientia of* Pakistan (Ullah et al. 2017) to over 88%, wb in *Eruca sativa* of Pakistan (Khan et al. 2016), showing a wide variation in water content that affects shelf life and nutritional density. High‐protein species like *Portulaca oleracea* (24.2 g/100 g) in Ethiopia (Aragaw et al. 2021) and *Rumex patientia* (24.4 g/100 g) in Pakistan (Ullah et al. 2017) stand out, indicating their potential as plant‐based protein sources. Similarly, high‐fat species such as *Monodora myristica* (53.4 g/100 g) in Cameroon suggest their utility as energy‐dense foods (Abdou Bouba et al. 2012).

Mineral content varies significantly as well, with some species like *Ceiba patendra* and *Vigna unguiculata* exhibiting extremely high calcium levels (4610 mg/100 g and 3357.3 mg/100 g, respectively), which are beneficial for bone health (Patricia et al. 2014). Iron concentrations are also remarkable in plants like *Malva neglecta* (110 mg/100 g) and *Piper umbellatum* (97.8 mg/100 g), supporting their use in addressing iron deficiency anemia. Zinc content is highly variable, with *Goldbachia laevigata* showing exceptionally high levels (34.1 g/100 g) in Pakistan (Khan et al. 2016). Overall, the systematic review emphasizes the significant potential of WEPs to contribute to nutritional security, particularly in resource‐limited settings. Their richness in essential nutrients makes them valuable in combating malnutrition, diversifying diets, and supporting sustainable food systems. Standardization and further research into bioavailability, antinutritional factors, and consumption safety are recommended to fully harness the benefits of these underutilized food resources.

Phytochemicals are naturally occurring, biologically active compounds in plants that offer therapeutic benefits beyond macronutrients and micronutrients. These secondary metabolites possess various biological properties, including antioxidant and antimicrobial activities, enzyme detoxification regulation, immune system modulation, inhibition of platelet aggregation, hormone metabolism regulation, and anticancer effects (Saxena et al. 2013). The most common phytochemicals in fruits and vegetables include vitamins C and E, carotenoids, flavonoids, polyphenols, and thiol (SH). Among these, polyphenolics are the most abundant secondary metabolites and are the primary antioxidant compounds (Berardini et al. 2005; Schieber et al. 2000). Likewise, certain WEPs in Ethiopia have been reported to contain significant phytochemicals.

WEPs not only provide nutritional benefits but also possess therapeutic properties due to their rich content of phytochemicals. As a result, they can be classified as functional foods that promote health and well‐being (Vanzani et al. 2011). These plants serve as valuable sources of pharmaceutical compounds, playing a significant role in drug development and the healthcare system (Omotayo and Aremu 2020). Additionally, they contribute to balanced diets and help enhance immunity against various diseases (Heinrich et al. 2016; Pieroni et al. 2008).

WEPs contain diverse secondary metabolites, including polyphenols, flavonoids, polysaccharides, and terpenoids, which have potential health‐promoting properties (García‐Herrera et al. 2014). Approximately 64% of the global population relies on traditional medicine for healthcare needs (Phondani et al. 2016). Traditional medicine encompasses knowledge and practices used to diagnose, prevent, and treat health conditions, developed over generations through observation and experience, either orally transmitted or documented (WHO 2000). The medicinal properties of plants were discovered over time through trial and error by early humans who sought remedies for ailments, pain, and injuries (Flatie et al. 2009).

A review of phytochemical data from WEPs across Africa and Asia reveals significant nutritional and health‐promoting potential due to their high contents of vitamin C, beta‐carotene, total phenolics, and flavonoids. Several Ethiopian species exhibit remarkably high vitamin C concentrations, such as *Carisa spinarum* (256.55 mg/100 g), *Dioscorea cayenensis* (259.33 mg/100 g), *Rubus apetalus* (294.19 mg/100 g), and *Syzygium guineense* (330.72 mg/100 g), highlighting their role as important natural sources of this essential antioxidant (Aragaw et al. 2021). Particularly outstanding is *Ximenia caffra* from Tanzania, which contains 358.82 mg/100 g of vitamin C, while *Rumex vesicarius* from Saudi Arabia also shows a high value at 253 mg/100 g (Mapunda and Mligo 2019; Kibar and Kibar 2017).

Beta‐carotene, a precursor of vitamin A and another potent antioxidant, is less widely reported but appears in notable amounts in species like *Cleome gynandra* (28.67 mg/100 g), *Trilepisium madagascariense* (24.92 mg/100 g), and *Vigna membranacea* (34.49 mg/100 g), mostly from Ethiopian flora (Yimer et al. 2023b). Meanwhile, total phenolic contents which are indicative of antioxidant capacity are extraordinarily high in several species. For example, *Aegle marmelos* from Thailand (8168 mg GAE/100 g) and *Moringa stenopetala* from Ethiopia (3900 mg GAE/100 g) stand out for their exceptionally rich phenolic profiles (Kubola et al. 2011; Dessalegn and Rupasinghe 2021). Others like *Ximenia americana* , *Ziziphus mauritiana* , and *Spondias pinnata* also exhibit strong phenolic contents, exceeding 2000 mg GAE/100 g (Lamien‐Meda et al. 2008; Kubola et al. 2011).

Flavonoid levels, which contribute to anti‐inflammatory and anti‐carcinogenic properties, are likewise abundant in several WEPs. *Aegle marmelos* again leads with 2168 mg CE/100 g, followed by *Carissa carandas* (1469 mg CE/100 g), *Moringa stenopetala* (1100 mg CE/100 g), and *Chrysophyllum cainito* (1117 mg CE/100 g), primarily from Thailand (Kubola et al. 2011). African plants such as *Ximenia caffra* (178.46 mg CE/100 g) and *Gardenia erubescens* (112.85 mg CE/100 g) also contribute meaningfully (Mapunda and Mligo, 2019; Jiru et al. 2023). Even lesser‐known species like *Goldbachia laevigata* and *Medicago polymorpha* from Pakistan demonstrate high flavonoid levels, suggesting potential for broader nutritional use (Khan et al. 2016). Overall, this broad phytochemical spectrum underscores the significance of WEPs as valuable sources of bioactive compounds. Their nutritional richness, particularly in antioxidants, supports their integration into local diets as functional foods, especially in regions where food insecurity and micronutrient deficiencies are prevalent. Promoting the use and conservation of these plants could contribute to improved public health and sustainable food systems.

WEPs distributed in Ethiopia should be efficiently utilized for various purposes. In addition, the traditional knowledge regarding the preparation and use of WEPs should be documented and supported with scientific evidence. Currently, various contributing factors are challenging the existence of essential WEPs. Thus, due attention should be given to preserving WEPs. Therefore, collaborative actions of policymakers of agriculture, health, and economy, and all influential and essential stakeholders are vital.

WEPs are crucial in rural and national economies by providing food, income, and resources for medicinal and industrial applications. These plants support livelihoods, contribute to food security, and offer opportunities for sustainable economic growth (FAO 2010). Many rural communities, especially in Africa and Asia, rely on WEPs for daily sustenance and as a source of revenue. For example, in Ethiopia, WEPs such as *Moringa stenopetala* and *Balanites aegyptiaca* are commonly harvested and sold in local markets, generating income for smallholder farmers and women‐led households (Getachew et al. 2013).

Beyond direct consumption, WEPs significantly reduce household food expenses since they require minimal inputs such as fertilizers and irrigation. This low cost of production makes them a cost‐effective food source, especially during droughts and food shortages, when cultivated crops may fail (Turner et al. 2011). Their resilience to harsh environmental conditions also ensures their availability in times of crisis, providing a natural food security buffer for vulnerable populations. In Ethiopia, WEPs like *Cordia africana* and *Dioscorea abyssinica* serve as emergency food supplies during a famine (Asfaw and Tadesse 2001).

WEPs also contribute to the *pharmaceutical industry*, as many species contain phytochemicals in traditional and modern medicine. Plants such as *Prunus africana* (African Cherry) are highly valued in the global medicinal market for their role in treating prostate disorders (Stewart 2003). Ethiopia's traditional medicine sector heavily depends on WEPs, which provide affordable healthcare alternatives to rural populations (Kassaye et al. 2006). The commercialization of medicinal WEPs has created new economic opportunities, with some species being exported for herbal medicine production.

WEPs in Ethiopia have significant practical implications for agriculture, health, and the economy, and their promotion holds considerable policy relevance. Agriculturally, WEPs contribute to diversification and resilience by offering drought‐tolerant, low‐input alternatives that are well‐adapted to local environments. Their integration into farming systems enhances agro‐biodiversity and supports sustainable land use, especially in marginal or degraded areas. From a health perspective, WEPs are a vital source of micronutrients, vitamins, and antioxidants, playing a critical role in combating malnutrition and food insecurity, particularly in rural and vulnerable communities. In addition, many WEPs are used in traditional medicine, supporting local healthcare systems and offering potential for inclusion in broader health strategies. Economically, WEPs provide opportunities for income generation through their collection and sale, particularly benefiting women and marginalized populations. The development of value chains around WEPs, including processing and marketing, can stimulate rural economies and create new livelihood options. Furthermore, the cultural and ecological value of WEPs can be leveraged to promote eco‐tourism and preserve indigenous food heritage. To fully realize these benefits, policy frameworks should integrate WEPs into national food security and nutrition strategies, support research and development for their conservation and domestication, empower community‐based management systems, and promote market access and value addition. By recognizing the multifaceted roles of WEPs, Ethiopia can enhance food sovereignty, environmental sustainability, and economic resilience.

## 5 Conclusion

WEPs play a significant role in Ethiopia's food security, nutrition, healthcare, and economic sustainability. The country's rich biodiversity, with 679 WEP species across 74 plant families, highlights the vast potential of these plants in addressing malnutrition, food shortages, and health challenges. Despite their widespread use by local communities, WEPs remain vastly underutilized in mainstream agriculture and scientific research. The global disparity between plant diversity and human utilization underscores the need to integrate WEPs into agricultural and food systems. While conventional crops dominate food production, WEPs provide essential nutrients, resilience against climate change, and medicinal properties. Prominent species such as *Syzygium guineense, Cordia africana, Carissa spinarum*, and *Ficus sur* have demonstrated high nutritional value and phytochemicals contributing to health benefits. However, limited scientific studies on their phytochemical composition, safety, and potential applications necessitate further investigation. To maximize the benefits of WEPs, interdisciplinary collaboration among researchers, policymakers, and local communities is essential. There is a need for systematic documentation of traditional knowledge, conservation efforts, and sustainable cultivation strategies. Additionally, promoting WEPs for commercial use can generate income for rural households while supporting biodiversity conservation. Recognizing and utilizing WEPs can contribute significantly to food security, healthcare, and sustainable economic development. Ensuring their preservation and efficient use requires immediate action through scientific research, policy support, and community engagement. By integrating WEPs into modern food systems, Ethiopia and the global community can harness their potential to improve nutrition, health, and environmental sustainability.

## 6 Future Prospectives

The future of WEPs in Ethiopia is promising, with significant potential to enhance food security, improve nutrition, support environmental sustainability, and contribute to economic development. Integrating WEPs into mainstream agriculture through domestication and controlled cultivation can diversify food production, reduce reliance on staple crops, and provide resilient food sources amid climate change. Advancing scientific research on the nutritional and medicinal properties of WEPs is crucial to validating their benefits, ensuring food safety, and exploring their potential applications in pharmaceuticals and nutraceuticals. Conservation efforts must also be prioritized, as many WEP species face deforestation, overharvesting, and land conversion threats. Establishing protected areas, implementing sustainable harvesting practices, and promoting agroforestry can help preserve these valuable resources.

Government policies and institutional support play a key role in promoting WEP utilization. Incorporating WEPs into national food security strategies, providing incentives for cultivation, and facilitating market access can encourage wider adoption. The commercialization of WEPs offers economic opportunities for rural communities by supporting value‐added industries such as herbal medicine, dried fruit processing, and organic food production. Expanding local and international markets for WEP‐based products can generate income and stimulate economic growth. Additionally, WEPs contribute to climate resilience by thriving in harsh environments, making them ideal candidates for sustainable land management and reforestation programs.

To preserve traditional knowledge surrounding WEPs, efforts must be made to document and integrate indigenous practices into modern agricultural and academic systems. Ethnobotanical studies, digital databases, and public awareness campaigns can help safeguard this knowledge for future generations. Ethiopia can harness the full potential of its diverse WEP species by fostering collaboration between researchers, policymakers, and local communities. Investing in sustainable utilization, scientific research, and commercial opportunities will ensure that WEPs remain vital for nutrition, health, and economic stability, contributing to long‐term national and global food security.

In addition, molecular and genetic analysis, phytochemicals profiling, and metabolomics aspects of WEPs should be investigated to fully understand their genetic diversity, adaptive traits, and biochemical compositions. Advanced molecular techniques such as DNA barcoding and genome sequencing can help identify and classify WEP species, facilitating their conservation and sustainable utilization. Genetic studies can also aid breeding programs to enhance desirable traits like drought resistance, disease tolerance, and improved nutritional content. Furthermore, a comprehensive analysis of the phytochemicals profiling of WEPs is essential to uncover their potential health benefits. Investigating phytochemicals and profiling them in WEPs, such as flavonoids, polyphenols, alkaloids, and antioxidants, can provide valuable insights into their therapeutic applications in traditional and modern medicine. Metabolomics, which studies the complete set of metabolites in plant species, can further elucidate the biochemical pathways responsible for nutrient synthesis and medicinal properties and should also be investigated for WEPs in Ethiopia. These approaches will strengthen the scientific validation of WEPs and create new opportunities for their integration into the pharmaceutical, nutraceutical, and functional food industries.

## 7 Limitations

This study has several limitations that should be acknowledged. The literature review was conducted using only two databases, which may have resulted in the omission of relevant studies available in other sources. Due to resource and access limitations commonly faced in low‐income countries, Scopus and Web of Science databases were not used in this systematic review. Moreover, as the focus is exclusively on WEPs of Ethiopia, the study lacks broader traditional and scientific insights concerning species that are not endemic to the country. This geographic limitation restricts the global applicability of the findings. Additionally, *publication bias* may have influenced the results, as studies with significant or positive outcomes are more likely to be published, potentially over‐representing favorable evidence. The *underreporting of gray literature* such as these s, reports, and non‐peer‐reviewed materials further contributes to potential gaps, particularly concerning lesser‐known or negative findings. Finally, *regional imbalances in data availability* may limit the representativeness and generalizability of the study, as certain areas may be underrepresented due to a lack of accessible or documented research. Publication bias, characterized by the preferential publication of studies reporting significant, positive, or novel findings while disregarding those with null or negative results, presents a critical concern in phytochemical and nutritional research, particularly in studies on WEPs. This bias can distort the scientific understanding of the true nutritional or medicinal value of these plants. Finally, the author also acknowledges the issue of *language bias*, a prevalent challenge faced by researchers worldwide. Language bias occurs when studies published in certain languages typically—English are—more likely to be indexed, cited, and considered in systematic reviews or meta‐analyses, while valuable research published in other languages is often overlooked. This can lead to an incomplete or skewed understanding of the nutritional and phytochemical composition of WEPs, particularly those studied in non‐English‐speaking regions.

## Author Contributions

**Sileshi Belew:** conceptualization (equal), data curation (equal), writing – original draft (equal), writing – review and editing (equal). **Gemmechu Hasen:** conceptualization (equal), investigation (equal). **Tilahun A. Teka:** conceptualization (equal), data curation (equal). **Sirawdink Fikreyesus Forsido:** conceptualization (equal), data curation (equal), investigation (equal), methodology (equal), supervision (equal), validation (equal). **Tamene Daba Rumicha:** conceptualization (equal), data curation (equal), writing – original draft (equal), writing – review and editing (equal).

## Ethics Statement

The authors have nothing to report.

## Consent

The authors provide consent to publish the manuscript.

## Conflicts of Interest

The authors declare no conflicts of interest.

## Data Availability

Data sets to support this are available upon reasonable request from the author.
